# Mini review: physically active schools as an arena for promoting health and cognition at the same time

**DOI:** 10.3389/fspor.2025.1532809

**Published:** 2025-05-02

**Authors:** Jennifer Liersch, Karin Eckenbach, Michael Pfitzner

**Affiliations:** Division of Sport Pedagogy and Subject-matter Teaching and Learning, University of Duisburg-Essen, Essen, Germany

**Keywords:** executive functions, physical activity, physical education, classroom, school sports, physically active breaks, movement games

## Abstract

Children and young adults spend a large part of their daily lives at school. Due to the increasingly critical physical and mental health of students, various concepts have been developed over the past decades to ensure that physical activity is implemented regularly and wherever possible in everyday school life. Although the relevance of these concepts is widely recognized, physical activities are often cancelled first when time is (suddenly) short. To secure cognitive components, low-movement core subjects are given preference over health-relevant physically active parts. However, there is empirical evidence that targeted integration of physical activity can improve students' cognitive performance, even when the amount of academic core subjects is reduced. The promotion of executive functions through movement has been demonstrated to be a useful approach. The findings of relevant studies are presented and discussed here in relation to different settings in everyday school life, including physical education, extracurricular school sports, and other learning areas. The aim is to demonstrate and justify ways of implementing physical activity in everyday school life and to promote the health and cognitive development of the students at the same time.

## 1 Introduction

In educational context, schools often fail to provide sufficient opportunities for physical activity (PA) and enjoyment of it, which is necessary for the healthy development of students. Although physical education (PE) is a compulsory subject in most countries, its absence is often accepted without resistance. A report by the European Commission indicates that PE has a relatively low status in Europe compared to other subjects (1). Over the past three decades, the number of hours of compulsory PE has been reduced in several European countries. Additionally, it is not uncommon for PE lessons to be cancelled at short notice in order to allow time for core subjects (2). Overall, the integration of PA into the school environment is often perceived more as a burden than an enrichment (3). In particular, the time aspect of implementation is seen as a hindrance (4).

These circumstances are problematic as they impede regular PA, which is crucial for students' health. It is undisputed that PA is associated with positive physical, psychosocial, and cognitive health (5). Schools, as collective institutions for all children and young adults, have the potential to establish PA in students' everyday lives and motivate them to engage in sports all their lives. Everyday school life could and should contribute to achieving the recommended 60 min of PA per day (6).

In addition to promoting health, promoting cognition provides another strong argument for the systematic implementation of PA in everyday school life. In a framework for the successful implementation of PA in schools, Chalkley et al. (7) emphasize that the simultaneous integration of education and health paradigms can strengthen acceptance of regular PA. In addition, research findings from the practical field justify the implementation of PA. This article aims to illustrate the connection between health-promoting PA and cognitive development. We ask to what extent cognitively beneficial effects can be achieved by including PA in everyday school life referring to empirical studies from the school. We focus on studies that concentrate on effects on the executive system. The relevance of the executive system is widely recognized, because of its relation to self-regulatory (8) and learning-related processes (9). Executive functioning is divided into the areas of inhibition (the ability to break automatic patterns of behavior and block out disturbing stimuli), cognitive flexibility (the ability to switch between different demands or adapt quickly), and updating (the ability to mentally process information stored short-term, e.g., to rearrange it) (10). Evidence from exercise neuroscience shows that especially cognitively demanding exercise interventions have positive effects on the executive system (11).

In contrast to previous reviews that take a broader view of cognitive performance and focus for example on academic performance in general [e.g., (12); Singh et al., 2019], we take a detailed look at a specific area (executive functions). At the same time, we consider all potential implementation scenarios in everyday school life and do not limit ourselves to a single area or rather exclude special areas [as was done, for example, in (13)]. Furthermore, we exclude clinical studies and measurement, as well as studies that also include interventions outside of the school environment [e.g., (11, 14–16)]. This mini-review exclusively considers longitudinal field studies whose results are valid in the authentic school setting.

To facilitate the presentation of the results, we adhere to the structural framework of PA, play, and sports from the German state of North Rhine-Westphalia (17). This framework encourages the systematic use of PA throughout the school context. Opportunities to integrate PA into everyday school life are divided into three pillars: PE, extracurricular school sports, and other learning areas or subjects. We analyzed existing literature regarding these pillars to determine their individual and combined beneficial impact.

## 2 Method

In line with the study aim and to distinguish it from existing studies (see Chapter 1), only certain studies were included in this mini-review. The search for suitable studies was based on several inclusion criteria. The findings presented refer to studies that:

• were designed as field studies, i.e., were integrated into everyday school life and implemented by the actors working there,
• assessed the executive system as an outcome measure,
• were designed as long-term intervention studies (no cross-sectional studies).

The review revealed various approaches based on empirical research. These approaches can be found for each of the three pillars of the structural framework. In total 13 sources were included.

## 3 Results

### 3.1 Physical education

There are two methods of implementing approaches in PE: Either by organizing lessons with specific and cognitively demanding content or by implementing movement games that challenge students both cognitively and physically. Schmidt et al. (18) conducted an intervention study to investigate the impact of two different teaching designs in PE lessons that differed in the cognitive demand of the movements and compared them with regular PE lessons. One group engaged in team games that incorporated cognitive demand and supplementary rules and the other group performed movement exercises with the objective of enhancing aerobic fitness while minimizing cognitive demand. The six-week intervention comprised 12 sessions of 45 min each, with two sessions per week. The study involved 181 pupils aged 10–12 in Switzerland. Inhibition, cognitive flexibility, and updating were measured. The results showed a significant improvement in cognitive flexibility in the group that participated in the team games compared to the control group and the other intervention group.

In contrast, cognitively demanding movement games are detached from the content of the lesson and can, therefore, be used more variably. A 20-week, controlled intervention study compared such specially designed movement games, which require executive functions to cope with the demands, according to the principles, with regular PE classes (19). The intervention consisted of two weekly sessions of 10–15 min each and was delivered to 197 German children aged 11–13. The results showed a significant improvement in inhibition in favor of the intervention group. It was particularly remarkable that children with weaker baseline scores and older children achieved greater improvement. Kolovelonis et al. (20) followed the same intervention idea with specially designed movement games. However, the intervention was shorter, lasting only 8 weeks, while the individual sessions were longer with 45 min. The study only measured inhibition. Still, an effect in favor of the intervention was also found for the 99 Greek children with an average age of 9 years who took part. In addition, a follow-up showed that the effects were still noticeable one month later.

Pesce et al. (21) and Lichtensteiger (22) also examined the implementation of cognitively demanding movement games in PE lessons, although with particular emphases. In the study conducted by Pesce et al. (21), the efficacy of a motor and coordination-demanding intervention design was investigated in Italian educational settings. The intervention was conducted over a six-month period, with children participating in a one-hour unit once a week. The units were led by external specialists and conducted outside the school context. The parameters measured included inhibition and updating. The results of the sample, comprising 460 children aged between 5 and 10, indicate a significant improvement in inhibition. Lichtensteiger (22) compared the effects of two types of movement games at German primary schools, fitness and coordination games. As part of the study, the children participated in the games, twice a week for 15–20 min at the beginning of PE lessons over a period of 22 weeks. The focus of the study was on inhibition and updating, with the sample (*n* = 282; age: 6–12 years) demonstrating an effect in both components in favor of both intervention groups compared to the control group.

The aforementioned points can be summarized as follows: Studies on physical education have shown that cognitively demanding movement games can improve children's executive functions, such as cognitive flexibility and inhibition. These games, which challenge both the body and the mind, have been found to enhance cognitive skills more effectively than traditional PE lessons. The benefits were particularly noticeable in children with weaker baseline cognitive abilities and persisted even after the interventions.

### 3.2 Extracurricular school sports

Some empirical studies can be found regarding the pillar of extracurricular school sports. Existing studies expanded the weekly school sports with additional sports programs. Crova et al. (23) implemented two additional sports lessons related to tennis, with the content divided between the promotion of basic motor skills and the development of tennis-specific skills. The study was conducted at two primary schools in Italy with 70 children aged between 9 and 10. While no effects were observed in the updating component, a comparison of overweight (37%) and non-overweight children indicated that the former benefited significantly more from the intervention in inhibition. Konijnenberg and Fredriksen (24) examined the impact of an additional daily PA lesson. In Norwegian primary schools, 1,173 children between the ages of 7 and 12 participated in physical activities and games implemented by teachers in various indoor and outdoor settings, including school playgrounds, gymnasiums, and hallways. The study only analyzed inhibition, and there were no intervention-related effects identified. The additional exercise program by van der Niet et al. (25) was conducted during the lunch break. In addition to moderate to intensive exercise, the program included cognitive challenges for the 99 Dutch pupils aged between 8 and 12. The intervention was conducted twice a week for a duration of 30 min, spanning a total of 22 weeks. All three components of the executive system were included in the measurements, but only inhibition and updating showed statistically significant improvement in comparison to the control group.

To sum up studies on extracurricular school sports have explored the effects of additional PA programs on children's executive functions. Some interventions, like tennis lessons and daily PA sessions, showed limited cognitive benefits. However, programs that combined exercise with cognitive challenges led to significant improvements in inhibition and updating compared to control groups.

### 3.3 Other learning areas and subjects

This section outlines various approaches to integrate movement-based programs into the classroom. These include full movement-based lessons or single learning components, as well as physically active breaks during a lesson.

De Greeff et al. (26) investigated the effects of physically active lessons on the executive functions of primary school children with an average age of 8 years. In two consecutive school years, teachers implemented a 22-week intervention program consisting of three 30-minute lessons per week. Each unit integrated an equal amount of mathematics and language content, combined with moderate to high-intensity PA. In a sample of 499 children, the three components of inhibition, cognitive flexibility, and updating were recorded, but no effects were identified. In their study, Mavilidi et al. (27) also identified that movement-based learning components integrated into English lessons had no impact on executive functioning. However, the intervention period was limited to six weeks.

Egger et al. (28) explored the potential of physically active breaks in the classroom. The study employed a between-subjects design with three experimental groups: a cognitive focus, a cognitively and physically active conceptualization, and a purely physically active implementation. Teachers at primary schools in Switzerland integrated the physically active breaks for ten minutes twice a day over a period of 20 weeks. Before and after the intervention period, all three components of the executive system were measured in 142 children aged 7–9. The results demonstrated significant improvement in cognitive flexibility in favor of the combined group, while inhibition and updating showed no intervention-related effects.

In a further intervention study, Mavilidi et al. (29) compared the effects of a program of pure physically active breaks with a program that combined such breaks with additional math exercises. The pure physically active breaks comprised physical activities such as squats or jumping jacks, while the combined physically active breaks demanded solving mathematical problems or answering questions while performing the physical activities. Both forms of physically active breaks were video-based. The physically active breaks had a length of two to three minutes and were integrated into mathematics lessons at Australian primary schools for a period of four weeks on three days a week. The breaks were implemented at the beginning and during the lesson. The study analyzed the components of inhibition and updating and reached the conclusion that the intervention had no effect on the two executive functions in the sample of 87 children with an average age of 9 years.

Overall, the following can be stated: Studies on integrating movement into classroom learning have explored various approaches, such as movement-based lessons, active learning components, and physically active breaks. Some interventions, like combining PA with subjects such as math and language, showed no significant effects on executive functions. However, one study found improvements in cognitive flexibility when physically active breaks were combined with cognitive tasks, while other approaches, like purely physically active breaks, did not lead to significant changes in executive functions.

### 3.4 Concepts

Two school concepts designed to encourage more PA in the everyday school environment were evaluated in Norway. These concepts combined different PA components. In the “Active School” program, Kvalø et al. (30) adapted three school elements that are no longer sedentary but physically active: homework, breaks and whole lessons. This increased PA at nine participating Norwegian schools from the regular 135 min to a total of 325 min. A total score for executive functions was measured in 449 children aged 9 and 10, and while there were tendencies, no significant effects were observed. When considered individually, however, the components demonstrated considerable improvement in the intervention group regarding updating. Aadland et al. (31) developed a similar concept in collaboration with teachers, comprising the same three components. In a larger sample of 1,129 children with an average age of 10 years, no overall effect was observed. Nevertheless, in analyses that considered the implementation of the intervention and referred to the test subjects who completed at least 80% of the intervention, there were minor statistically significant effects regarding executive functions in favor of the intervention group.

The concepts presented are aimed at increasing PA. They showed mixed results on executive functions. While both programs increased PA through active homework, breaks, and lessons, they found no significant overall effects. However, some improvements in executive functions, particularly updating, were observed for students who participated fully in the interventions.

## 4 Discussion

**Figure 1:**
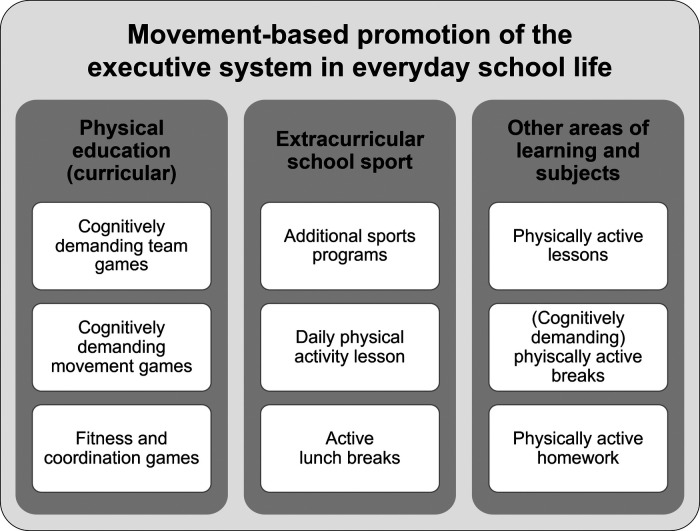
Intervention approaches for movement-based promotion of the executive system in everyday school life, divided into curricular physical education, extracurricular school sport and other areas of learning and subjects.

Referring to the structural framework of PA, play, and sports made it possible to systematically uncover different forms of implementation of PA in everyday school life. Studies were identified that met the previously defined search criteria and related to the three pillars of the framework. Regarding the three pillars, different intervention approaches have been established in research to promote the executive system. It is possible to derive a wide range of application within an active school day. Referring to the framework, the various approaches identified are presented as a diagram in Figure 1.

Overall, most positive effects showed for inhibition control. One possible explanation for this is that movement-based situations in which inhibition skills are required are very authentic and easy to integrate into the classroom. An example is the use of simple visual or acoustic signals to call for or prohibit certain reactions (cf. “Simon Says”).

Fewer positive effects were found in the pillar “Other learning areas and subjects” compared to the other pillars. Further differentiation in this area and the development of additional implementation options are therefore recommended.

The significance of the results is limited by three main facts: First, only long-term studies were included in the analysis. Short-term studies with acute effects were disregarded. Second, there is limited comparability among the referenced studies because the study designs differ in terms of the test instruments used, the intervention content, and the duration of the interventions. Third, the referenced studies look at the individual pillars almost exclusively in isolation. Still, in some studies, a more holistic approach is already pursued in the sense of PA-friendly schools: concepts that go beyond the individual pillars are developed and evaluated. The studies by Kvalø et al. (30) and Aadland et al. (31) can be cited as examples of such an approach. In each of these studies, various measures to promote PA in everyday school life were developed and then evaluated regarding the executive system. The cross-sectional study by (32) follows a similar approach: In this study, different forms of PA are examined in relation to inhibitory control. 340 children with an average age of 8.6 years completed a self-report on their PA and sports activities during the school day. According to the results, unstructured PA during break time was associated particularly with good inhibition performance.

It is recommended to invest in overarching, holistic concepts in the future to expand the effects of PA on health and cognition. The practical advice of de Greeff et al. (11) should be considered: Exercise interventions should occur regularly and over several weeks.

## 5 Conclusion

Many solutions for the implementation of movement-based promotion of the executive system have already been proposed. Overall, it can be said that a PA-orientated school day does not only promote an active lifestyle and benefit health. It also promotes executive functions—an area of central importance for cognitive performance.

Schools need to disrupt sedentary habits as often as possible and implement movement in a targeted manner. This can take the form of cognitively demanding physically active breaks in the classroom or targeted cognition-enhancing content in PE lessons, for example. In addition, students should be motivated to spend break times actively, for example by designing playgrounds in a stimulating way.

The study situation can still be described as heterogeneous. There is an acute need for development in relation to other learning areas and subjects. At the same time, holistic concepts need to be developed, implemented, and evaluated regarding a school culture of movement.
